# The inner logic of digital anxiety: a Self-Determination Theory perspective on the motivational transformation of Fear of Missing Out

**DOI:** 10.3389/fpubh.2026.1741670

**Published:** 2026-02-25

**Authors:** Han-Lin Hsiung, Hui-Ling Hu

**Affiliations:** 1 Department of Business Administration, University of Kang Ning, Taipei, Taiwan; 2 Institute of Creative Design and Management, National Taipei University of Business, Taipei, Taiwan

**Keywords:** autonomous motivation, competence, Fear of Missing Out (FoMO), prosocial orientation, Self-Determination Theory (SDT), social media addiction, social media use, social support

## Abstract

**Introduction:**

Social media addiction among university students has been increasingly associated with psychological vulnerabilities, particularly Fear of Missing Out (FoMO). Drawing on Self-Determination Theory (SDT) and Social and Emotional Learning (SEL), this study examines the associations between autonomous motivation, social media competence, social support, FoMO, and social media addiction, and investigates the moderating role of prosocial orientation.

**Methods:**

A questionnaire survey was conducted among 422 university students in Taiwan. Structural Equation Modeling (SEM) was employed to examine the hypothesized associations among autonomous motivation, competence, social support, FoMO, and social media addiction. A moderation analysis was conducted to test the interaction effect of prosocial orientation.

**Results:**

Autonomous motivation, social media competence, and social support were significantly negatively associated with FoMO. FoMO was significantly positively associated with social media addiction. In addition, prosocial orientation significantly moderated the relationship between FoMO and social media addiction, such that the positive association was stronger among individuals with higher levels of prosocial orientation.

**Discussion:**

The findings highlight the psychological associations between motivational resources, FoMO, and addictive social media behaviors. By integrating SDT and SEL perspectives, this study provides a more comprehensive understanding of digital anxiety and underscores the context-dependent role of prosocial orientation in digitally mediated social interactions.

## 1 Introduction

In the digital era, social media has become deeply embedded in university students’ daily lives, serving as a primary channel for information acquisition, social interaction, and self-expression. While these platforms offer substantial benefits, growing evidence indicates that excessive and uncontrolled use is associated with social media addiction, which has been linked to anxiety, depressive symptoms, impaired attention, and reduced psychological well-being (1, 2). These findings suggest that social media addiction is not merely a behavioral issue of overuse but reflects deeper psychological and emotional vulnerabilities.

Among the psychological factors associated with problematic social media use, Fear of Missing Out (FoMO) has been consistently identified as a central risk-related construct. FoMO refers to persistent anxiety arising from concerns that one may miss rewarding experiences that others are having (3). Prior studies have shown that individuals experiencing higher levels of FoMO tend to engage in more frequent checking behaviors and exhibit stronger tendencies toward addictive social media use (4, 5). These findings highlight FoMO as a key psychological correlate linking social comparison, emotional insecurity, and excessive media engagement.

To advance understanding of the psychological correlates underlying FoMO and social media addiction, this study adopts an integrative perspective drawing on Self-Determination Theory (SDT) and Social and Emotional Learning (SEL). SDT emphasizes the role of basic psychological needs in shaping motivation and well-being, whereas SEL focuses on individuals’ emotional regulation and interpersonal competencies. Integrating these perspectives allows FoMO to be conceptualized as a central motivational–emotional construct associated with psychological need frustration, insufficient social–emotional competencies, and maladaptive digital behaviors.

Despite the growing literature on FoMO and social media addiction, existing studies predominantly rely on single-path explanations and rarely integrate motivational need structures with trainable emotional and social resources. Moreover, limited attention has been paid to moderating factors that may shape the association between FoMO and addictive behaviors. In particular, prosocial orientation—closely related to social awareness and relationship skills—has theoretical relevance for understanding individual differences in responses to FoMO within digital environments, yet empirical evidence remains limited.

Addressing these gaps, the present study proposes a dual-layer theoretical framework that integrates SDT and SEL to examine how autonomous motivation, competence, and social support are associated with FoMO, how FoMO is associated with social media addiction, and whether prosocial orientation moderates this relationship. By conceptually situating FoMO at the intersection of motivational need satisfaction and social–emotional regulation, this study aims to enrich theoretical understanding of digital anxiety and provide practical insights for promoting healthier social media engagement among university students.

## 2 Literature review

### 2.1 Theoretical foundations

#### 2.1.1 Social and Emotional Learning

Social and Emotional Learning (SEL) has evolved beyond an educational intervention framework to become an important perspective for understanding individuals’ emotional regulation, interpersonal functioning, and behavioral adjustment. Rather than focusing solely on academic outcomes, SEL emphasizes individuals’ capacities to recognize emotions, regulate behavior, navigate social relationships, and make responsible decisions in complex environments (6, 7).

Recent studies have increasingly suggested that deficiencies in social–emotional competencies may be associated with maladaptive behaviors in digital contexts. In particular, insufficient self-management and emotional regulation have been linked to problematic patterns of media use, including excessive engagement and compulsive checking behaviors (8). From this perspective, social media addiction can be viewed as a behavioral manifestation of emotional dysregulation and interpersonal insecurity within highly stimulating digital environments.

Although SEL has been widely applied in school-based intervention research, its potential as a theoretical framework for explaining university students’ digital behaviors remains underdeveloped. Existing studies often emphasize the outcomes of SEL programs rather than its explanatory value for understanding psychological mechanisms underlying technology-related anxiety and addiction. This gap highlights the need to position SEL not merely as an educational tool, but as a conceptual lens for examining how emotional and interpersonal competencies shape individuals’ responses to digital media environments.

#### 2.1.2 Self-Determination Theory

Self-Determination Theory (SDT) provides a well-established motivational framework for understanding human behavior and psychological well-being. The theory posits that individuals possess three fundamental psychological needs—autonomy, competence, and relatedness—which are essential for sustaining intrinsic motivation and healthy functioning (9, 10). Prior SDT research suggests that higher levels of psychological need satisfaction are associated with greater self-regulation and psychological resilience, whereas need frustration is often associated with compensatory behavioral tendencies.

In the context of digital media use, SDT has been increasingly employed to understand patterns of social media engagement related to psychological compensation. Lower levels of autonomous motivation are often associated with diminished control over their online behavior, while low competence can heighten uncertainty and dependence on constant information updates. Similarly, unmet relatedness needs have been associated with a greater tendency to seek connection and validation through online interactions rather than offline relationships (11). Taken together, prior SDT-based research suggests that social media addiction is closely associated with underlying motivational vulnerabilities, rather than being solely a matter of excessive use.

Despite its strong explanatory power, prior research applying SDT to digital behavior has primarily focused on direct associations between need satisfaction and media use outcomes. Less attention has been paid to how motivational need frustration interacts with individuals’ emotional and social competencies. As a result, SDT-based explanations of social media addiction remain incomplete when detached from the emotional regulation and interpersonal skill dimensions emphasized by complementary psychological frameworks.

#### 2.1.3 Integrating SEL and SDT

While SEL and SDT originate from different theoretical traditions, they address complementary aspects of human functioning. SDT focuses on the structural foundations of motivation through universal psychological needs, whereas SEL highlights individuals’ capacities to regulate emotions and manage social relationships in everyday contexts. Integrating these perspectives allows for a more comprehensive understanding of digital behavior, in which motivational need frustration and social–emotional competence deficits are jointly associated with maladaptive outcomes. Although Social and Emotional Learning (SEL) is discussed as a complementary perspective for understanding emotional regulation and interpersonal functioning in digital contexts, the present study does not directly operationalize SEL competencies as core latent constructs. Instead, SDT serves as the primary theoretical framework guiding construct operationalization and the development of research hypotheses, while SEL is employed as an interpretive lens to enrich the discussion of emotional and social regulatory processes.

Within this integrative framework, social media addiction can be conceptualized as being associated with both unmet psychological needs and insufficient emotional regulation in digitally saturated environments. This combined perspective provides a theoretical foundation for examining the associations between motivational vulnerabilities, digital anxiety, and maladaptive engagement patterns. Building on this integration, the following sections focus on FoMO as a central psychological construct that is closely associated with these processes.

### 2.2 Fear of Missing Out and social media addiction

Building on the theoretical foundations outlined in Section 2.1, this section focuses on empirical empirical evidence examining the associations between Fear of Missing Out (FoMO) and social media use behaviors, particularly problematic and addictive patterns. Existing studies have consistently shown that individuals with higher levels of FoMO tend to engage more frequently with social media platforms, exhibit compulsive checking behaviors, and experience greater difficulty disengaging from online social interactions (4, 5, 12).

Within digital environments characterized by constant connectivity and real-time social comparison, FoMO has been associated with heightened sensitivity to social cues, such as notifications, updates, and peer activities. These characteristics are closely related to patterns of excessive monitoring and repetitive engagement with social media, which have been identified as core features of social media addiction (2). Empirical findings suggest that FoMO is positively associated with addictive social media use across different populations, including adolescents and university students (5, 13).

Rather than conceptualizing FoMO as a formally tested mediating variable, prior research positions it as a central psychological construct that is closely associated with both emotional insecurity and maladaptive digital engagement. FoMO represents a focal experiential state reflecting individuals’ anxiety about social exclusion and missed opportunities, and has been consistently associated with problematic patterns of social media use. Accordingly, the present study adopts FoMO as a core construct within the proposed framework to examine its association with social media addiction among university students.

### 2.3 Autonomous motivation in social media use, competence, and social support

While Section 2.1 introduced the core principles of Self-Determination Theory (SDT), this section narrows the focus to empirical studies examining how SDT-related motivational factors are associated with FoMO and digital media behaviors. Research grounded in SDT suggests that variations in autonomous motivation, competence, and relatedness are closely linked to individuals’ psychological experiences in online contexts (10, 14).

Autonomous motivation in social media use has been associated with greater self-regulation and more intentional engagement, whereas controlled or externally driven use is more closely related to individuals’ psychological experiences (11, 13). Individuals who perceive their online engagement as self-endorsed and value-consistent tend to report lower levels of anxiety related to social comparison and online evaluation. Lower levels of such anxiety have been negatively associated with FoMO.

Similarly, perceived competence in managing social media platforms is associated with individuals’ confidence in navigating information flows, regulating attention, and coping with information overload. Empirical evidence indicates that higher levels of digital competence are associated with lower uncertainty and reduced anxiety related to missing social information. These patterns are consistent with a negative association between competence and FoMO-related experiences.

In addition, social support—closely related to the SDT need for relatedness and emphasized within Social and Emotional Learning (SEL) frameworks—has been consistently linked to psychological well-being and adaptive coping. Individuals with stronger offline social support networks tend to report lower dependence on online social validation and reduced vulnerability to FoMO (14). These findings highlight the importance of integrating motivational and social–emotional resources when examining FoMO in digital contexts.

Taken together, prior research suggests that autonomous motivation, competence, and social support are meaningfully associated with FoMO-related experiences in online environments. Rather than proposing causal pathways, the present study builds on these associations to examine how SDT- and SEL-related factors are linked to FoMO and social media addiction within an integrated conceptual framework.

### 2.4 The relationship between autonomous motivation in social media use, competence, and social support with FoMO

Building on the conceptualization of FoMO as a central psychological construct (see Section 2.2), this study examines the associations between social media–related psychological resources and individuals’ FoMO experiences. As discussed in Section 2.3, autonomous motivation, competence, and social support are associated with individual differences in social media engagement patterns and the interpretation of social information in digital environments.

Individuals with higher levels of autonomous motivation in social media use tend to report more goal-directed and intentional engagement patterns, and lower levels of anxiety related to social comparison and online evaluation. In contrast, lower autonomous motivation has been associated with greater reliance on constant connectivity and higher levels of FoMO.

*H1:* Autonomous motivation in social media use is negatively associated with FoMO.

Similarly, social media competence reflects individuals’ perceived ability to manage information and interactions effectively. Higher perceived competence is associated with more selective engagement and lower uncertainty, whereas lower competence has been associated with greater concerns about informational delay and social exclusion.

*H2:* Social media competence is negatively associated with FoMO.

Finally, social support is commonly associated with emotional reassurance and a stable sense of belonging, which are linked to lower reliance on continuous online monitoring. When perceived social support is lower, individuals tend to report greater sensitivity to social cues and updates, as well as higher levels of FoMO.

*H3:* Social support is negatively associated with FoMO.

### 2.5 FoMO and social media addiction

As discussed in Section 2.2, FoMO is conceptualized as a central psychological construct that is closely associated with motivational vulnerabilities and difficulties in emotional regulation within digital environments. Prior research has consistently shown that individuals reporting higher levels of FoMO also report more frequent checking behaviors and higher levels of excessive social media use (4, 5).

Empirical evidence further indicates a strong positive association between FoMO and problematic or addictive patterns of social media use among university students. Rather than implying causal processes, these findings suggest that FoMO is a salient psychological correlate of social media addiction within digitally mediated social contexts. Accordingly, the present study examines the association between FoMO and social media addiction, as specified in the following hypothesis.

*H4:* FoMO is positively associated with social media addiction.

### 2.6 The moderating role of prosocial orientation

In addition to risk-related psychological factors, individual differences may be associated with variations in the relationship between FoMO and social media addiction. Prosocial orientation reflects individuals’ tendencies toward empathy, cooperation, and maintaining interpersonal relationships, which may be related to how they experience and respond to FoMO-related anxiety.

On the one hand, higher prosocial orientation has been associated with greater perceived emotional support and more meaningful social connections, which may be linked to lower levels of distress in social contexts. On the other hand, strong relational sensitivity has also been associated with greater responsiveness to social expectations and perceived obligations in digital contexts. Taken together, these considerations suggest that prosocial orientation may moderate the relationship between FoMO and social media addiction.

*H5:* Prosocial orientation moderates the relationship between FoMO and social media addiction.

**Figure 1:**
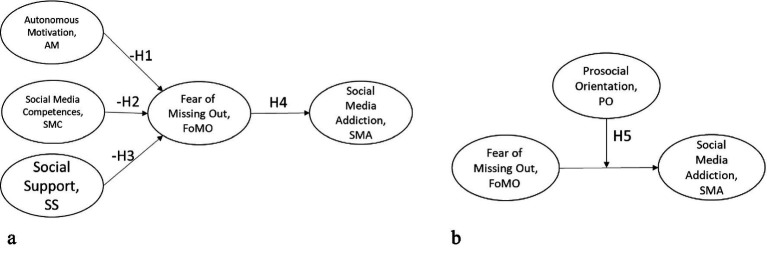
**(a)** Model A – path model. **(b)** Model B – moderation model.

Figures 1a,b illustrates the conceptual research framework of this study. Model A represents the hypothesized path model tested using the main structural model, whereas Model B represents the moderation model examined separately using an interaction term approach. Autonomous motivation in social media use, social media competence, and social support are specified as key psychological resources that are hypothesized to be negatively associated with Fear of Missing Out (FoMO). FoMO is conceptualized as a central psychological construct that is positively associated with social media addiction. In addition, prosocial orientation is modeled as a moderating variable that conditions the strength of the association between FoMO and social media addiction. The framework reflects the hypothesized directional associations examined in this study.

## 3 Research methodology

### 3.1 Research subjects and data collection

The subjects of this study are university students in Taiwan, who are one of the primary groups using social media and are in a critical period of exploring self-identity and establishing social relationships. The specific research subjects include university students from different grades, majors, and backgrounds to ensure the breadth and generalizability of the research results. The study utilized the researcher’s network connections and distributed the electronic questionnaire link through social media groups of university students from different regions and schools between June 1, 2024, and June 30, 2024, to recruit students willing to participate in the study. A total of 422 valid questionnaires were collected, meeting the sample size recommendation of 300–500 by statisticians (15, 16).

### 3.2 Questionnaire design

The questionnaire for this study is composed of two main sections: basic demographic information and Likert-scale items measuring the core research constructs. The demographic section collects information on participants’ gender, age, and major. The second section includes measurement items related to autonomous motivation in social media use, social media competence, social support, FoMO, social media addiction, and prosocial orientation. To ensure theoretical rigor, the design of each construct were grounded in established literature. The construct of autonomous motivation in social media use was developed based on SDT proposed by Deci and Ryan (9), which emphasizes intrinsic motivation and autonomous motivation as key determinants of self-regulated behavior. The items reflect individuals’ reasons for engaging in social media, such as enjoyment, personal meaning, and value congruence. It should be noted that the items primarily capture autonomous motivation—reflecting intrinsically motivated or value-congruent engagement with social media—rather than autonomous motivation need satisfaction per se. This operationalization is consistent with prior SDT-based studies that focus on motivational regulation rather than basic need fulfillment. Similarly, the measurement of social media competence was derived from SDT’s conceptualization of competence, representing individuals’ perceived effectiveness and mastery in their interactions with digital environments (17–19). The construct of social support was designed with reference to House’s (20) classical social support theory, which highlights the critical role of emotional and instrumental support in promoting psychological well-being. The dimension of FoMO was adapted from Przybylski et al. (3), defining FoMO as the anxiety individuals experience when fearing the loss of meaningful or enjoyable experiences that others may be having. Finally, the construct of prosocial orientation was based on the work of Eisenberg et al. (21, 25), which describes the tendency to engage in behaviors that benefit others—such as empathy, cooperation, and altruism—in social interactions. Overall, the questionnaire integrates validated theoretical frameworks from SDT, social support theory, and prosocial behavior research to comprehensively examine psychological factors associated with social media use and addiction. The full list of measurement items for all constructs is provided in Appendix A for reference. Accordingly, all core constructs in this study are operationalized based on SDT-related motivational and resource-based concepts, while SEL is employed as a complementary interpretive perspective rather than being directly operationalized as competency measures.

### 3.3 Data analysis

This study employs Structural Equation Modeling (SEM) as the primary statistical analysis method to examine the structural relationships among the study variables. SEM integrates Confirmatory Factor Analysis and regression-based path analysis, allowing for the simultaneous examination of multiple relationships among latent constructs. The analysis process consists of two main stages: the Measurement Model and the Structural Model. First, the Measurement Model specifies the relationships between observed indicators and their corresponding latent variables and is evaluated using Confirmatory Factor Analysis (CFA). Second, the Structural Model examines the hypothesized directional associations among the latent variables within the proposed conceptual framework. Finally, a path diagram is used to visually represent the SEM framework, illustrating the specified paths and relationships among variables to facilitate interpretation of the overall model structure.

## 4 Research results

### 4.1 Analysis of demographic information

A total of 422 valid questionnaires were collected. Among the respondents, 219 were male, accounting for 51.9% of the sample. Participants aged 18–20 years constituted the largest age group (*n* = 224, 53.1%). In terms of academic background, students from medical and healthcare-related majors represented the largest proportion of the sample (*n* = 145, 34.4%). Detailed demographic information is presented in Table 1.

**Table 1:** Analysis of demographic information.

Variable	Category	Frequency distribution	Percentage
Gender	Female	203	48.1%
	Male	219	51.9%
Age	18–20 years old	224	53.1%
	20–22 years old	152	36.0%
	Above 22 years old	46	10.9%
Major	Humanities and social sciences	57	13.5%
	Other disciplines	50	11.8%
	Business and management	82	19.4%
	Science and engineering	26	6.2%
	Medical and healthcare	145	34.4%
	Arts and design	19	4.5%
	Tourism and leisure	43	10.2%

### 4.2 Convergent validity

Following the criteria proposed by Fornell and Larcker (22), convergent validity was assessed using factor loadings, composite reliability (CR), average variance extracted (AVE), and Cronbach’s *α*. The results show that all factor loadings ranged from 0.750 to 0.917, exceeding the recommended threshold of 0.7. Composite reliability values ranged from 0.872 to 0.925, indicating satisfactory internal consistency. The AVE values ranged from 0.652 to 0.743, all above the 0.5 criterion, and Cronbach’s α coefficients ranged from 0.867 to 0.913. Together, these results indicate that the measurement model demonstrates adequate convergent validity. Detailed results are presented in Table 2.

**Table 2:** Convergent validity analysis.

Constructs	Items	Factor loadings	Cronbach’s alpha	Composite reliability (CR)	Average variance extracted (AVE)
Autonomous motivation	AM1	0.860	0.879	0.925	0.655
	AM2	0.750			
	AM3	0.853			
	AM4	0.737			
	AM5	0.839			
FoMO	FoMO1	0.861	0.915	0.917	0.745
	FoMO2	0.824			
	FoMO3	0.897			
	FoMO4	0.872			
	FoMO5	0.861			
Prosocial orientation	PO1	0.809	0.867	0.872	0.652
	PO2	0.811			
	PO3	0.861			
	PO4	0.763			
	PO5	0.789			
Social media addiction	SMA1	0.762	0.900	0.906	0.716
	SMA2	0.862			
	SMA3	0.864			
	SMA4	0.894			
	SMA5	0.842			
Social media competences	SMC1	0.761	0.883	0.941	0.676
	SMC2	0.826			
	SMC3	0.816			
	SMC4	0.892			
	SMC5	0.812			
Social support	SS1	0.882	0.913	0.922	0.743
	SS2	0.917			
	SS3	0.804			
	SS4	0.837			
	SS5	0.866			

AM, autonomous motivation; FoMO, Fear of Missing Out; PO, prosocial orientation; SMA, social media addiction; SMC, social media competences; SS, social support.

### 4.3 Discriminant validity

Discriminant validity was assessed using the Average Variance Extracted (AVE) criterion proposed by Fornell and Larcker (22). The results show that, for most constructs, the square root of the AVE exceeds the correlations with other constructs, indicating adequate discriminant validity. Detailed results are presented in Table 3.

**Table 3:** Discriminant validity analysis.

Constructs	Autonomous motivation	FoMO	Prosocial orientation	Social media addiction	Social media competences	Social support
Autonomous motivation	**0.809**					
FoMO	−0.426	**0.863**				
Prosocial orientation	0.452	−0.394	**0.807**			
Social media addiction	−0.299	0.679	−0.382	**0.847**		
Social media competences	0.674	−0.473	0.593	−0.418	**0.822**	
Social support	0.631	−0.456	0.631	−0.293	0.702	**0.862**

The bold values on the diagonal represent the square root of the Average Variance Extracted (AVE) for each construct.

### 4.4 Goodness of fit

GOF (Goodness of Fit) is an overall measure of the model’s fit, calculated as $\mathrm{GOF}=\sqrt{\bar{\mathrm{AVE}}}\times \sqrt{\bar{R^{2}}}$ . According to Vinzi et al. (23), a GOF value of 0.1 indicates weak fit, 0.25 indicates moderate fit, and 0.36 indicates strong fit. The result of this study shows a GOF value of 0.507, indicating a strong fit.


$$\mathrm{GOF}=\sqrt{\bar{\mathrm{AVE}}}\times \sqrt{\bar{R^{2}}}=\sqrt{0.686\times 0.374}=0.507$$

### 4.5 Path analysis

The results of the structural model indicate that autonomous motivation in social media use is negatively associated with FoMO (*β* = −0.138, SD = 0.052, *t* = 2.655, *p* = 0.008). Social media competence is also negatively associated with FoMO (*β* = −0.238, SD = 0.066, *t* = 3.610, *p* < 0.001), as is social support (*β* = −0.202, SD = 0.060, *t* = 3.390, *p* = 0.001). In addition, FoMO is positively associated with social media addiction (*β* = 0.612, SD = 0.034, *t* = 18.085, *p* < 0.001).

Beyond statistical significance, the relative magnitudes of the path coefficients provide descriptive information regarding the relative strength of these associations. Among the three antecedents of FoMO, social media competence shows the strongest negative association (*β* = −0.238), followed by social support (*β* = −0.202), whereas autonomous motivation in social media use exhibits a comparatively weaker, though still significant, negative association (*β* = −0.138). This pattern indicates that students’ perceived ability to effectively manage social media environments shows a stronger negative association with FoMO than motivational autonomy alone.

**Figure 2:**
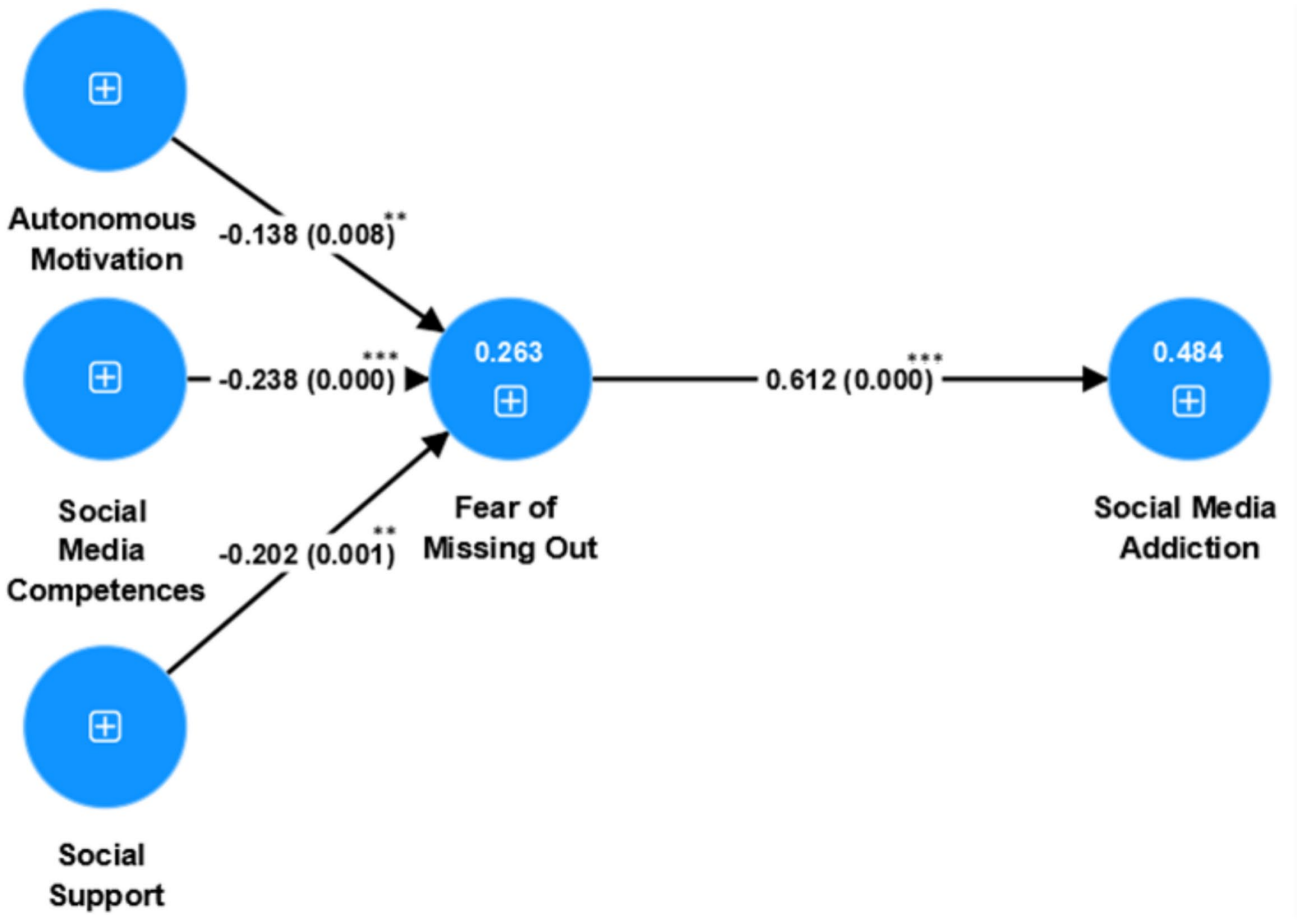
PLS-SEM statistical model diagram.

Furthermore, the strong positive association between FoMO and social media addiction indicates that higher levels of FoMO co-occur with higher levels of excessive and compulsive social media use. Taken together, these findings highlight the potential relevance of digital competence and social support in understanding FoMO and its association with problematic social media use. Table 4 presents the detailed results of the path analysis, and Figure 2 illustrates the structural model.

**Table 4:** Path analysis.

Path relationship	Original sample (O)	Standard deviation (STDEV)	*T* statistics (|O/STDEV|)	*p* values
Autonomous motivation → FoMO	−0.138	0.052	2.655	0.008
Social media competences → FoMO	−0.238	0.066	3.610	0.000
Social support → FoMO	−0.202	0.060	3.390	0.001
FoMO → Social media addiction	0.612	0.034	18.050	0.000

### 4.6 Moderation effect

SmartPLS provides established procedures for testing moderation effects through the construction of interaction terms (24). Following this approach, moderation was examined using a separate analysis in which an interaction term between FoMO and prosocial orientation was specified. Consistent with standard practice, this moderation analysis was conducted independently from the main structural model, which focuses on the hypothesized direct associations among the core constructs.

**Figure 3:**
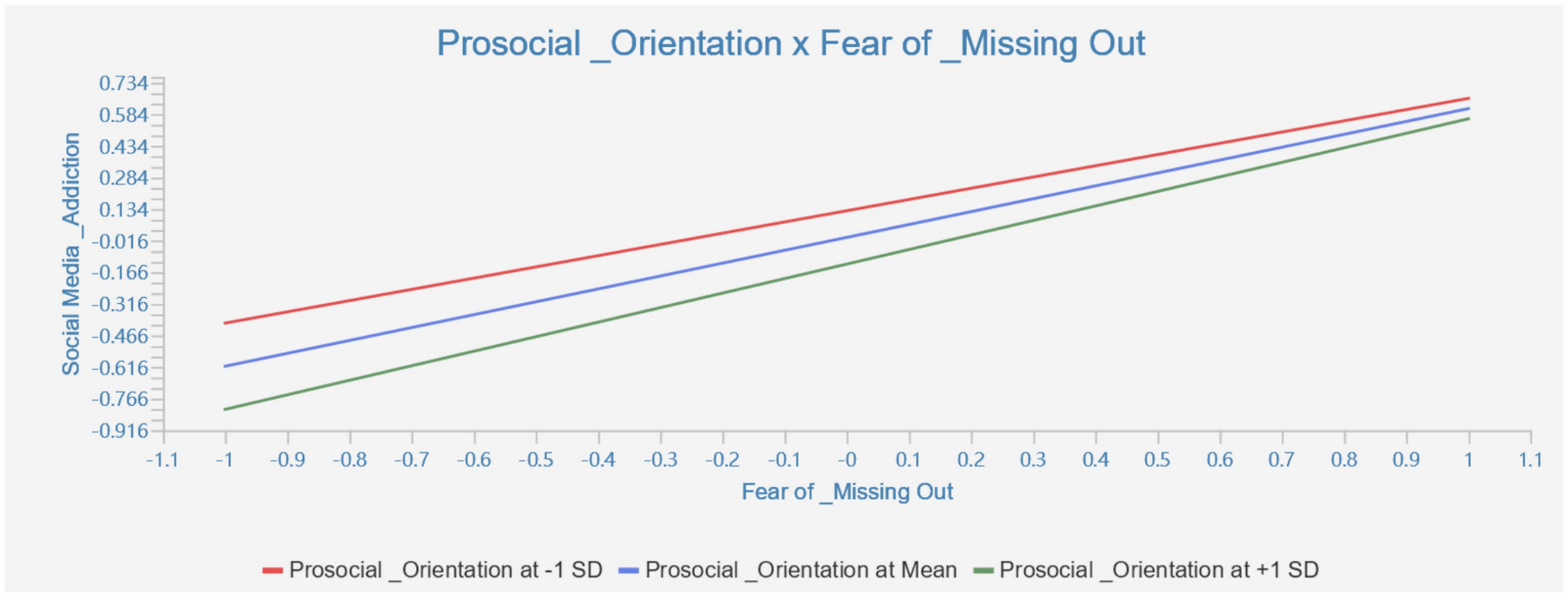
Moderation effect diagram of prosocial orientation × FoMO on social media addiction.

The results show that the interaction term between FoMO and prosocial orientation is significantly associated with social media addiction, indicating a statistically significant moderation effect. For clarity of presentation, the moderation effect is reported separately from the main path model. Detailed results of the moderation analysis are presented in Table 5, and the interaction pattern is illustrated in Figure 3.

**Table 5:** Moderation effect analysis.

Path relationship	Path coefficient	Standard deviation	*t* value	*p* value
Prosocial orientation × Social media addiction → Social media addiction	0.078	0.038	2.051	0.040

## 5 Conclusion and discussion

### 5.1 Research conclusions

#### 5.1.1 Associations between autonomous motivation, competence, social support, and FoMO

The results of this study indicate that autonomous motivation, social media competence, and social support are significantly negatively associated with university students’ Fear of Missing Out (FoMO). These findings are broadly consistent with key perspectives in Self-Determination Theory (SDT) and Social and Emotional Learning (SEL), which emphasize the importance of psychological need satisfaction and social–emotional resources for maintaining psychological balance and well-being (9, 10).

From the perspective of SDT, autonomous motivation reflects the extent to which individuals perceive their behaviors as self-endorsed and aligned with internal values. Higher levels of autonomous motivation in social media use are associated with greater self-regulation and reduced reliance on external validation, which may be relevant for understanding its negative association with FoMO. University students reporting a stronger sense of autonomy and meaning in their online engagement also tend to report lower vulnerability to social comparison pressures and FoMO-related anxiety to social comparison pressures and the anxiety associated with missing out on others’ experiences (3).

Similarly, social media competence—conceptually related to the need for competence in SDT—is negatively associated with FoMO. Students with higher perceived competence in managing social media platforms may feel more capable of selectively engaging with content, filtering information, and regulating their online behaviors. Such perceived efficacy and control have been associated with lower uncertainty and reduced anxiety related to missing information or social interactions.

In addition, social support is negatively associated with FoMO, underscoring the relevance of relatedness needs and SEL principles. Emotional and instrumental support from family, friends, and peers are commonly associated with greater fulfillment of students’ social needs in offline contexts, which is linked to lower reliance on online social validation. Consistent with prior research, stronger real-life social support networks are associated with greater psychological security and lower levels of FoMO (14, 24).

Taken together, these findings support an integrated SDT–SEL perspective by demonstrating that autonomous motivation, competence, and social support are closely associated with lower levels of FoMO. Rather than implying causal effects, the results highlight meaningful psychological associations that help clarify how motivational and social–emotional factors are linked to FoMO among university students.

#### 5.1.2 Association between FoMO and social media addiction

The results further indicate that FoMO is significantly positively associated with social media addiction. This finding is consistent with prior research suggesting that individuals who experience higher levels of FoMO tend to report more frequent and compulsive patterns of social media use (4, 5).

From an SDT perspective, FoMO may reflect experiences related to unmet psychological needs—particularly relatedness and autonomy—which have been discussed in prior research in connection with compensatory patterns of social media engagement. Students who perceive gaps in social connection or self-expression may also report higher levels of FoMO, which have been associated with increased monitoring of social media content.

From the SEL perspective, difficulties in emotional regulation and self-management may be related to stronger FoMO–addiction associations. Students who report greater difficulty managing anxiety related to social comparison or perceived social exclusion also tend to report more repetitive checking behaviors and problematic patterns of use. Although the present study does not establish causal pathways, the observed association underscores FoMO as an important psychological factor associated with social media addiction within the proposed framework.

#### 5.1.3 Moderating role of prosocial orientation

The findings indicate that prosocial orientation significantly moderates the relationship between FoMO and social media addiction. Specifically, the positive association between FoMO and social media addiction is stronger among individuals with higher levels of prosocial orientation, suggesting an amplifying rather than buffering pattern of moderation.

Prosocial orientation is typically characterized by empathy, cooperation, and a strong concern for maintaining interpersonal relationships (24, 25). While these traits are generally regarded as beneficial in offline and relational contexts, the present findings suggest that in digitally mediated environments, they may be associated with heightened sensitivity to social expectations and perceived obligations. Under conditions of higher FoMO, prosocially oriented individuals may report greater sensitivity to social expectations and perceived obligations, which has been associated with stronger tendencies toward addictive social media use.

This pattern highlights the contextual dependency of prosocial traits in digital settings. Rather than functioning as a protective factor, prosocial orientation appears to be associated with a stronger FoMO–social media addiction relationship. These findings support a nuanced, double-edged interpretation of prosocial orientation, suggesting that traits typically considered adaptive may be associated with greater vulnerability to problematic media use under conditions of heightened social anxiety.

### 5.2 Theoretical contributions

This study makes several theoretical contributions to the literature on social media addiction and digital well-being. First, it conceptually integrates Self-Determination Theory (SDT) and Social and Emotional Learning (SEL) to provide a more comprehensive framework for understanding psychological correlates of FoMO and social media addiction. By jointly considering motivational needs and social–emotional competencies, the study extends existing research that has often examined these perspectives in isolation.

Second, this study positions FoMO as a central psychological construct within an integrated SDT–SEL framework. Rather than conceptualizing FoMO as a formally tested mediating variable, the findings highlight its role as a focal psychological factor that is closely associated with motivational need–related experiences and addictive patterns of social media use. This conceptual positioning helps clarify FoMO’s importance in linking motivational and emotional processes to digital behaviors without overstating causal or mediational claims.

Third, the study contributes to the literature by identifying prosocial orientation as a contextual moderator that strengthens the association between FoMO and social media addiction. This finding challenges the predominantly one-dimensional view of prosocial orientation as uniformly protective and underscores its complex, context-dependent role in digitally mediated social interactions. By showing that prosocial traits may be associated with greater vulnerability to addictive behaviors under certain conditions, the study opens new avenues for theoretical exploration of social motivation in digital environments.

### 5.3 Managerial and practical implications

The findings of this study offer several practical implications for educational institutions, mental health practitioners, and policy makers concerned with promoting healthy digital engagement among university students. First, the negative associations between autonomous motivation, competence, social support, and FoMO suggest that initiatives aimed at enhancing students’ self-regulation, digital literacy, and offline social resources may be beneficial. Educational programs that emphasize reflective social media use, goal-aligned engagement, and information management skills could support healthier patterns of use.

Second, given the positive association between FoMO and social media addiction, early identification of FoMO-related anxiety may be relevant for informing prevention-oriented support efforts. Counseling services and student support systems may consider incorporating FoMO-related assessments and interventions that focus on emotional regulation, self-management, and coping with social comparison.

Finally, the moderating role of prosocial orientation underscores the relevance of balanced approaches when encouraging social engagement and altruistic behaviors. While fostering empathy and cooperation remains valuable, practitioners should also emphasize boundary setting, digital self-care, and mindful connectivity. Encouraging students to maintain healthy limits in online responsiveness may be helpful in reducing the likelihood that prosocial tendencies become associated with excessive or compulsive social media use.

### 5.4 Sampling considerations and implications for interpretation

Despite the contributions of this study, several sampling-related limitations should be acknowledged. First, although the sample consists of university students in Taiwan, a notable proportion of the respondents (approximately 34.4%) are from medical and healthcare-related majors. Students in these disciplines often experience relatively high academic pressure, frequent information exposure, and intensive peer coordination through digital platforms. As a result, their patterns of social media use and psychological responses—particularly regarding Fear of Missing Out (FoMO)—may differ from those of students in other academic fields. This disciplinary concentration may therefore introduce a potential disciplinary bias and limit the generalizability of the findings to students in less academically intensive or differently structured programs.

Before discussing general methodological limitations, we first address sampling-specific considerations that are directly relevant to the interpretation of the present findings. Despite the contributions of this study, several limitations related to sampling characteristics should be acknowledged. First, a notable proportion of the sample (approximately 34%) consisted of students majoring in medical and healthcare-related fields. Students in these disciplines may exhibit higher levels of academic pressure, information sensitivity, and reliance on digital communication for academic coordination and peer support. As a result, their patterns of social media use and psychological responses to online information may differ from those of students in other academic fields. Although medical and healthcare majors represent an important segment of the university population, this disciplinary concentration may limit the generalizability of the findings to students in less academically intensive or differently structured programs.

Second, the recruitment strategy relied on social media groups targeting university students, which may introduce a degree of self-selection bias. Students who are more active on social media platforms are more likely to encounter and respond to online surveys, potentially leading to an overrepresentation of frequent social media users. Consequently, the observed levels of FoMO and social media addiction may be higher than those found in the general university student population. This sampling characteristic suggests that the results should be interpreted as reflecting psychological mechanisms among relatively active social media users, rather than serving as prevalence estimates applicable to all students.

Importantly, these sampling characteristics do not undermine the internal validity of the proposed theoretical relationships. The primary objective of this study was to examine the associative mechanisms linking social media–related psychological resources, FoMO, and social media addiction, rather than to estimate population-level prevalence. Nevertheless, future research could adopt stratified or multi-stage sampling strategies, recruit participants through offline channels, or conduct cross-disciplinary comparisons to enhance representativeness and further validate the robustness of the proposed model.

### 5.5 Research limitations and future directions

Despite its contributions, this study has several limitations that warrant consideration. First, the sample was limited to university students in Taiwan, which may constrain the cross-cultural generalizability of the findings. Future research could conduct cross-cultural comparative studies to examine whether the observed associations hold across different cultural and social contexts. Second, the research model primarily focused on autonomous motivation, competence, social support, FoMO, and prosocial orientation, while other potential influencing factors—such as personality traits, family background, and academic stress—were not included. Future studies could incorporate these variables to provide a more comprehensive understanding of factors associated with social media addiction. Third, this study adopted a cross-sectional design, which limits the ability to establish causal relationships among variables. Future research could employ longitudinal or experimental designs to further examine the temporal ordering and dynamic relationships among the constructs. Finally, as this study relied on self-reported questionnaires, there may be risks of common method bias and social desirability effects. To enhance validity, future studies are encouraged to adopt multi-method approaches, such as behavioral data analysis, digital trace tracking, or qualitative interviews, to triangulate findings and strengthen the robustness of the conclusions.

## Data Availability

The datasets generated and/or analyzed during the current study are publicly available in the Zenodo repository at: https://doi.org/10.5281/zenodo.13844060.
