# Supplementary material for: The inner logic of digital anxiety: a Self-Determination Theory perspective on the motivational transformation of Fear of Missing Out
Source: Front Public Health. 2026 Feb 25;14:1741670. doi: 10.3389/fpubh.2026.1741670 (PMC12975565; doi:10.3389/fpubh.2026.1741670)
Supplement: Supplementary file 1 [file Supplementary_file_1.docx]

### **Appendix A. Measurement Items**

All items were measured using a Likert-type scale ranging from 1 (strongly disagree) to 7 (strongly agree).

#### **Table A1. Autonomous Motivation in Social Media Use**

| Item Code | Measurement Item | Source |
| --- | --- | --- |
| AM1 | I use social media because it makes me feel happy and joyful. | Deci and Ryan (2000) |
| AM2 | When using social media, I feel that I am free to choose and control my actions. |  |
| AM3 | I use social media to express my thoughts and feelings. |  |
| AM4 | I use social media because I decide to do so, not because others expect me to. |  |
| AM5 | I use social media because it makes me feel fulfilled and meaningful. |  |

#### **Table A2. Social Media Competence**

| Item Code | Measurement Item | Source |
| --- | --- | --- |
| SMC1 | When using social media, I feel that I am able to learn and apply new skills and knowledge. | Ryan and Deci (2000) |
| SMC2 | My interactions on social media allow me to showcase my expertise and talents. |  |
| SMC3 | I can effectively solve problems and achieve goals on social media. |  |
| SMC4 | Using social media makes me feel like a capable and confident person. |  |
| SMC5 | I can easily master the various functions and operations on social media. |  |

#### **Table A3. Social Support**

| Item Code | Measurement Item | Source |
| --- | --- | --- |
| SS1 | I am able to receive emotional support from friends and family on social media. | House (1983) |
| SS2 | I can get practical help from friends and family on social media. |  |
| SS3 | When I need advice, I can find someone on social media who offers useful suggestions. |  |
| SS4 | I feel that I have a reliable social network on social media. |  |
| SS5 | When interacting with friends and family on social media, I feel their care and support. |  |

#### **Table A4. FoMO**

| Item Code | Measurement Item | Source |
| --- | --- | --- |
| FMO1 | When I can't check social media, I worry about missing important news or events. | Przybylski et al. (2013) |
| FMO2 | When I see my friends sharing interesting activities on social media, I feel like I missed out on those activities. |  |
| FMO3 | If I don't frequently check social media, I worry about being out of the loop. |  |
| FMO4 | I feel anxious when I can't participate in activities mentioned by my friends on social media. |  |
| FMO5 | I frequently check social media because I don't want to miss anything. |  |

#### **Table A5. Social Media Addiction**

| Item Code | Measurement Item | Source |
| --- | --- | --- |
| SMA1 | I often spend more time on social media than I originally planned. | Przybylski et al. (2013) |
| SMA2 | If I can't use social media, I feel anxious or irritable. |  |
| SMA3 | I have neglected other important activities or responsibilities because of social media use. |  |
| SMA4 | I have tried multiple times to reduce the time I spend on social media but have been unsuccessful. |  |
| SMA5 | I use social media in all my free time, even in social situations, I can't stop checking it. |  |

#### **Table A6. Prosocial Orientation**

| Item Code | Measurement Item | Source |
| --- | --- | --- |
| PO1 | I actively help my friends solve the problems they encounter on social media. | Eisenburg, Fabes, and Spinrad (2006) |
| PO 2 | I often share helpful information and resources with others on social media. |  |
| PO 3 | I comfort and support friends who are facing difficulties on social media. |  |
| PO 4 | I am willing to spend time participating in public welfare activities or charity projects on social media. |  |
| PO 5 | When friends seek help on social media, I respond immediately. |  |
